# Can creative productivity be *both* positively *and* negatively correlated with psychopathology? Yes!

**DOI:** 10.3389/fpsyg.2014.00455

**Published:** 2014-05-20

**Authors:** Dean Keith Simonton

**Affiliations:** Department of Psychology, University of California, DavisDavis, CA, USA

**Keywords:** creativity, psychopathology, productivity, genius, Lotka's Law

Empirical research on the hypothesized relation between creativity and psychopathology must take care to frame the question very carefully. If a person's creativity is defined by the output of creative products, then the empirical association can be either positive or negative, depending on how that association is specified. On the one hand, individuals who make at least one creative contribution to a domain may exhibit lower risk of psychopathology than those who never do. On the other hand, among those individuals who contribute one or more creative products, those who contribute the most creative products may have higher risk of psychopathology than those who contribute the fewest creative products. These two hypotheses can both be empirically confirmed because the cross-sectional distribution of creative output is described by a highly skewed inverse power function known as Lotka's Law (Lotka, 1926). That is, the number of individuals producing *n* creative products is proportional to 1/*n*^2^ (Egghe, 2005). Given this skewed distribution, the risk rate can easily increase as a linear function of creative productivity even though the overall risk rate is strikingly lower than in the general population.

To illustrate, suppose that the following Lotka function holds for a particular creative domain: *f*(*n*) = 100/*n*^2^. Then the lowest creative output is 1 and the highest 10. Let us also assume that the risk of some psychopathology increases as a positive linear function of *n*. In particular, we might specify the risk as *R*(*n*) = −0.100 + 0.100^*^n. According to this hypothesized function, the risk increases from *R*(1) = 0, for the lowest level of creative output, to *R*(10) = 0.90, for the highest level of creative output. It follows from the cross-sectional distribution that (a) nearly two-thirds (i.e., about 65%) will have zero risk of psychopathology and (b) the average risk for all individuals contributing one or more creative products is only 0.09 (or 9%). The latter figure is not only one tenth of the risk hypothesized for the most prolific creator, but also presumably noticeably smaller than would likely hold in the population of individuals who made no creative contributions to a domain. For instance, it might hold that *R*(0) = 0.46 (based on Kessler et al., 2005), a figure fivefold higher.

This treatment can be generalized beyond this specific illustration. Whenever *R*(1) << *R*(0), that is, the risk rate is much lower among the one-hit creative individuals, then it would still be possible to have *R*(*n*) increase with increases in creative productivity *n*. This increase does not even have to be linear, for a positive monotonic relation will have the same effect, yielding the inequalities *R*(1) < *R*(2) < *R*(3) < … *R*(*n* − 1) < *R*(*n*). In fact, the creativity-psychopathology relation in the literary and visual arts may be accurately described in this manner, and even the function for philosophers is very close to positive monotonic (Simonton, 2014b).

Consequently, researchers can find both positive and negative associations depending on which part of the distribution is actually sampled in their investigation. For example, creative geniuses can be more at risk than are their far less prolific or innovative colleagues. This expectation would explain the higher rates of psychopathology often found in historiometric research (Simonton, 2014a). In contrast, psychometric studies will more likely sample much less eminent creators who enjoy higher mental health, creating an apparent contradiction when none exists.

Naturally, it is reasonable to ask *why* this paradoxical finding might actually appear. Possible explanations fall into two categories. First, the cognitive and personality *antecedents* of genius-level creativity may put the individual at increased risk for psychopathological symptoms. For instance, higher creativity may require greater cognitive disinhibition, an inclination also associated with tendencies toward psychopathology (Carson, 2014). Second, a highly prolific and creative career may have *consequences* that can threaten mental health, such as increased criticism and even hostility in the reception of those products. It may be no accident that positive creativity-psychopathology relationships have most often been found in low-consensus domains where immediate appreciation by colleagues or audiences is by no means guaranteed, such as the expressive arts (Simonton, 2014b). The struggling and neglected artist is proverbial.

Ultimately, these possible outcomes and potential interpretations must be addressed by empirical research, but that research must have a more complex understanding of the questions asked.

## Conflict of interest statement

The author declares that the research was conducted in the absence of any commercial or financial relationships that could be construed as a potential conflict of interest.
